# Fatal intracardiac and pulmonary arterial thromboembolic damage following ABO-incompatible living donor liver transplantation for autoimmune hepatitis: A case report

**DOI:** 10.1097/MD.0000000000024298

**Published:** 2021-01-15

**Authors:** Won Kyu Choi, Junghan Kim, Ho Joong Choi, Sang Hyun Hong, Min Suk Chae

**Affiliations:** a Department of Anesthesiology and Pain Medicine, Uijeongbu St. Mary's Hospital; b Department of Anesthesiology and Pain Medicine, St. Vincent's Hospital; c Department of Surgery, Seoul St. Mary's Hospital; d Department of Anesthesiology and Pain Medicine, Seoul St. Mary's Hospital, College of Medicine, The Catholic University of Korea, Seoul, Republic of Korea.

**Keywords:** ABO incompatible living donor liver transplantation, autoimmune hepatitis, splenectomy, thromboembolism

## Abstract

**Rationale:**

We present the case of a patient with autoimmune hepatitis who suffered fatal intracardiac and pulmonary arterial thromboembolic complications after ABO-incompatible living donor liver transplantation (ABOi LDLT) with splenectomy.

**Patient concerns:**

A 46-year-old female (blood type B^+^) with autoimmune hepatitis and hepatitis B carrier status underwent elective ABOi LDLT. The donor liver was from a 51-year-old male living donor (blood type A^+^). A splenectomy was performed without bleeding complications. Intraoperatively, the patients hemodynamic condition was acceptable, with no evidence of thromboembolism on transesophageal echocardiography (TEE).

**Diagnosis:**

Postoperatively, her platelet count increased from 15.0 to 263.0 (× 10^9^/L) and thromboelastographic parameters indicated hypercoagulable state. She suffered acute circulatory collapse, respiratory distress and, eventually, a decline in mental status. The attending physicians in the intensive care unit (ICU) immediately performed resuscitation.

**Interventions:**

The patient underwent emergency exploratory surgery. Intraoperatively, hypotension, bradycardia and arrhythmia developed, together with high central venous pressure. Assessment of cardiac structure and function using rescue TEE incidentally identified multiple, huge thromboembolic clots in the cardiac chambers; therefore, the patient underwent cardiac thromboembolectomy, including cardiopulmonary bypass with hypothermia therapy.

**Outcomes:**

Due to severe cardiac and respiratory distress, the patient required venoarterial extracorporeal membrane oxygenation (VAECMO) in the operating room and ICU. Despite continuous resuscitation in the ICU and maintenance of VAECMO, she suffered severe hypotension and massive bleeding that eventually led to death.

**Lessons:**

In patients with autoimmune hepatitis, risk factors for thromboembolism should be rigorously controlled during the peak period of reactive thrombocytosis after ABOi LDLT with splenectomy.

## 1 Introduction

Autoimmune hepatitis is a chronic inflammatory liver disease, in which unknown factors trigger a T-cell-mediated immune activation.^[1]^ Patients with autoimmune liver diseases, including autoimmune hepatitis, primary biliary cholangitis and primary sclerosing cholangitis, account for up to 24% of liver transplantations (LTs).^[2]^ but among those who are waitlisted the mortality rate is high, at 15%.^[3]^ To address the shortage of liver donors, ABO-incompatible living donor liver transplantation (ABOi LDLT) is an emerging option for patients with end-stage liver disease.^[4]^ With improvements in desensitization management, including plasmapheresis and immunosuppression regimens, the graft survival rate in ABOi LDLT has improved greatly.^[5]^ Desensitization measures include splenectomy, given the spleens function in the production and storage of antibodies, B cells and plasma cells as well as in blood filtration, phagocytosis, red blood cell destruction, antigen uptake and hemopoiesis. However, splenectomy increases the risk of complications, including infection, portal vein thrombosis and bleeding.^[6,7]^ In addition, because ABO blood antigens are widely expressed on organ/tissue cellular surfaces, patients undergoing ABOi LDLT are more susceptible to immune-response-related complications, such as arterial thrombus, bile duct injury, rejection, sepsis, and graft failure.^[8]^

Here we describe the case of a patient with autoimmune hepatitis who suffered eventually fatal intracardiac and pulmonary arterial thromboembolic insults after undergoing ABOi LDLT with splenectomy. A legally authorized representative – her husband – has signed informed consent for publication of the anonymous case report. We carried out the study after obtaining approval from Ethics Committee at the Seoul St. Mary's Hospital (KC20ZISI0248) on April 16, 2020.

## 2 Case report

A 46-year-old female (height: 168.3 cm; weight: 53.6 kg; blood type B^+^) with liver-biopsy-proven autoimmune hepatitis (defined in accordance with the International Autoimmune Hepatitis Group criteria)^[9]^ and hepatitis B carrier status underwent elective ABOi LDLT. Her MELD (model for end-stage liver disease) score was 18 points, and she was being continuously treated with prednisolone and antiviral medication (Viread; Gilead, Foster City, CA, USA). Her laboratory findings were as follows: hemoglobin, 7.8 g/dl; white blood cell count, 4.12 × 10^9^/L; creatinine, 0.93 mg/dl; albumin, 3.0 g/dl; aspartate and alanine aminotransferase, 64 U/L and 30 U/L, respectively; total bilirubin, 2.97 mg/dl, and lactic acid 2.0 mmol/L. Her coagulation values included an international normalized ratio (INR) of 2.2, activated partial prothrombin time of 57.7 second, and platelet count of 17.0 × 10^9^/L. Abdominal computed tomography revealed advanced liver cirrhosis, partial thrombus in the main portal vein, moderate ascites, and marked splenomegaly and splenorenal shunt. Preoperative desensitization and immunosuppression were performed according to the ABOi LDLT protocol,^[10]^ and consisted of a single dose of intravenous rituximab (375 mg/m^2^ body surface area) administered 2 weeks before surgery (which reduced CD marker levels from 6.1%–0.1%) and multiple plasmapheresis (total 51 units of fresh frozen plasma [FPP]), to achieve an isohemagglutinin titer of ≤1:8 (Table 1).

**Table 1:** Serial clinical changes in hemodynamic and coagulation variables during the perioperative period.

		ABOi LDLT with splenectomy				Exploratory laparotomy			Cardiac thrombectomy with CPB			
	Preoperative findings	Preanhepatic phase	Anhepatic phase	Neohepatic phase	ICU	Before CPR	CPR	After CPR	Before CPB	CPB	After CPB	ICU with VA ECMO
		Routine TEE				Rescue TEE						
Vital signs												
SBP (mm Hg)	120	107	114	130	53	91	65	62	61		52	70
DBP (mm Hg)	70	56	57	55	21	39	50	33	28		41	40
HR (beats/minutes)	80	74	88	83	95	40		62			110	50
CVP (mm Hg)		10	4	7		30						
Blood product transfusion												
Washed PRBC (Blood type O^+^)	3 units	10 units	3 units	25 units	10 units							
FFP (Blood type AB^+^)	51 units	5 units		5 units								
SDP (Blood type AB^+^)		1 unit										
Coagulation variables												
1. Laboratory values												
Platelet count (10^9^/L)	17.0	19.0	18.5	15.0	263.0	81.0						33.0
INR	2.2	1.7	2.4	2.1	1.1	1.8						5.0
aPTT (sec)	57.7	35.7	94.4	80.4	30.2	43.0						120.0
Fibrinogen (mg/dL)		94.0	60.0	54.0								20.0
Antithrombin III (%)		39.5	26.9	26.0								7.9
D-dimer (mg/L FEU)		5.5	13.9	10.9								35.2
FDP (mcg/ml)		15.3	38.0	26.5								80.0
2. Kaolin-based thromboelastographic values												
Reaction time (minutes)					1.8 minutes							
K value (minutes)					0.3 minutes							
Alpha-angle (°)					88 °							
Maximal amplitude (mm)					122 mm							
Lysis time (%)					0%							

ABOi LDLT = ABO incompatible living donor liver transplantation, ALT = alanine aminotransferase, aPTT = activated partial thrombin time, AST = aspartate aminotransferase, BNP = brain natriuretic peptide, CPB = cardiopulmonary bypass, Cryo = cryoprecipitate, CVP = central venous pressure, DBP = diastolic blood pressure, FDP = fibrinogen degradation product, FFP = fresh frozen plasma, HR = heart rate, ICU = intensive care unit, INR = international normalized ratio, PRBC = packed red blood cell, SBP = systolic blood pressure, SDP = single donor platelets, T. bilirubin = total bilirubin, TEE = transesophageal echocardiography, VA ECMO = venoarterial extracorporeal membrane oxygenation, WBC = white blood cell.

The piggyback method was employed during LT, using the right hepatic lobe of a 51-year-old male living donor (blood type A^+^, donor-recipient weight ratio: 1.91%, steatosis 9.83%) and reconstructing the middle hepatic vein such that the segmental hepatic veins (from liver segments V and VIII) of the recipient were anastomosed with the middle hepatic vein of the donor. The hepatic vessels, including the hepatic vein, portal vein, and hepatic artery, were serially anastomosed followed by biliary ductal reconstruction. The patency of hepatic blood flow was evaluated using spectral Doppler ultrasonography. The patient underwent simultaneous splenectomy, without bleeding complications due to severe thrombocytopenia or collateral shunt. Intraoperative management was performed with balanced anesthesia and hemodynamic homeostasis, with appropriate circulatory adjustment. In addition, a vasopressor was administered under multiple hemodynamic monitoring, including transesophageal echocardiography (TEE; Vivid S5 cardiovascular ultrasound; GE Healthcare, Milwaukee, WI, USA) and laboratory measurements. Transfused blood products consisted of 10 units of washed packed red blood cells (PRBCs; blood type O^+^), 5 units of FFP (blood type AB^+^) and 1 unit of single donor platelets (SDPs; blood type AB^+^). An acceptable hemodynamic status was achieved during surgery, including the absence of postreperfusion syndrome^[11]^ or evidence of cardiac or pulmonary thromboembolism, as determined by TEE. The endotracheal tube was removed in the operating room without respiratory complications. The patient was then transferred to the intensive care unit (ICU) for continued monitoring.

**Figure 1:**
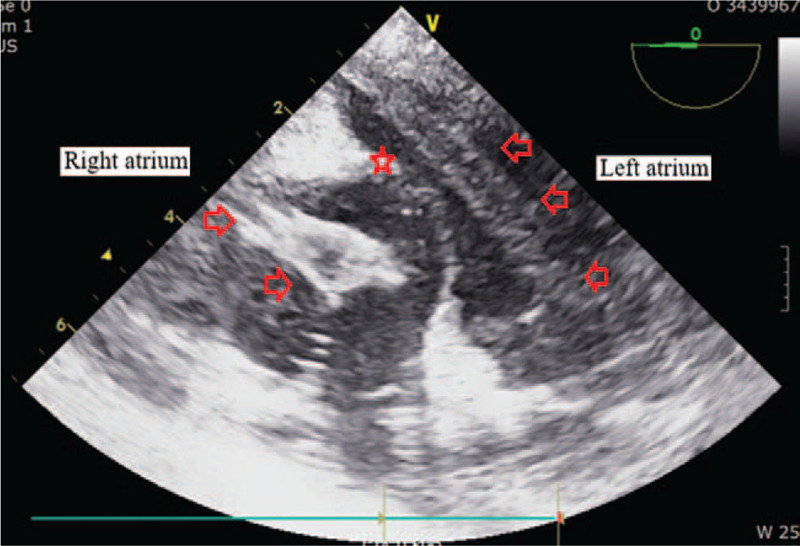
Multiple, huge thromboembolic clots in the right and left atria, seen on transesophageal echocardiography. Arrows point to the thromboembolic clots, and the star indicates the left-shifted atrial wall.

**Figure 2:**
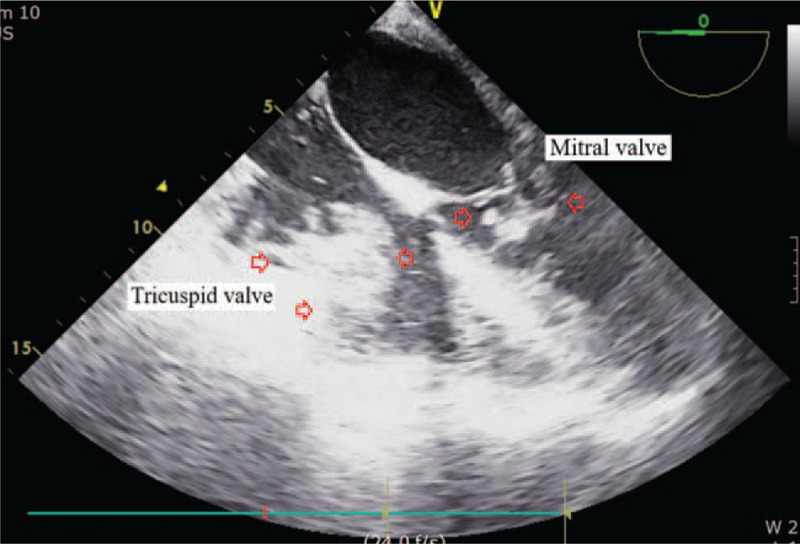
Multiple, huge thromboembolic clots on the tricuspid and mitral valves, seen on transesophageal echocardiography. Arrows point to the thromboembolic clots.

**Figure 3:**
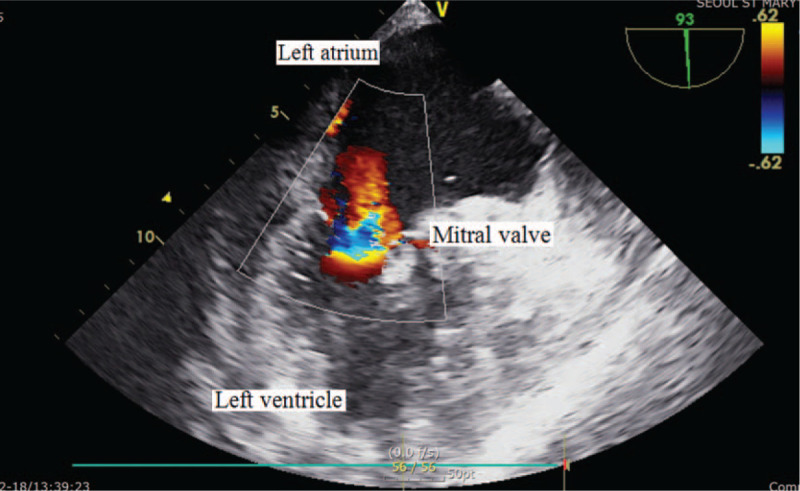
Severe mitral regurgitation seen on transesophageal echocardiography.

**Figure 4:**
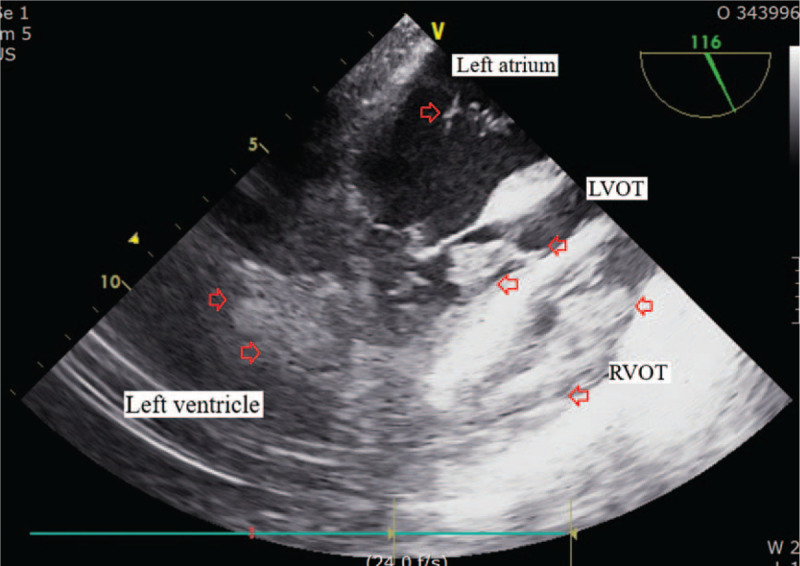
Huge and multiple thromboembolic clots in the left atrium and ventricle, left ventricular outflow tract and right ventricular outflow tract shown by transesophageal echocardiography. Empty arrows indicate thromboembolic clots.

**Figure 5:**
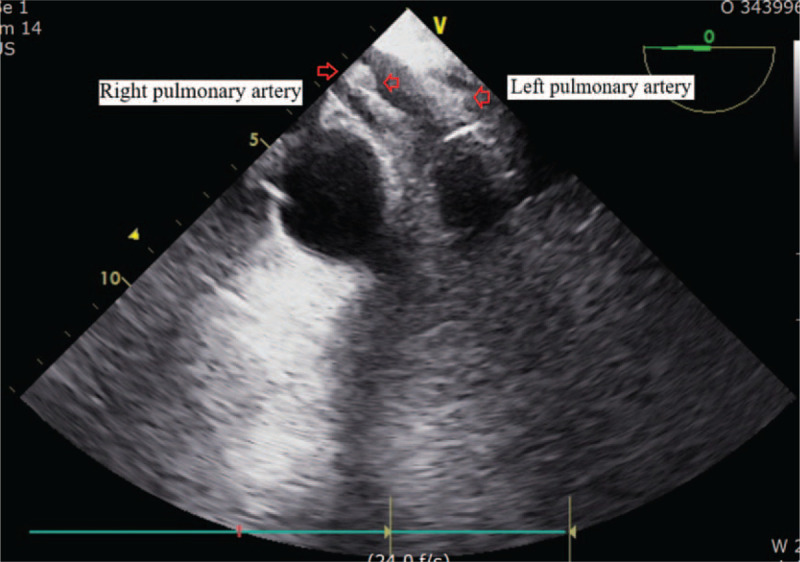
Multiple, huge thromboembolic clots in the right and left pulmonary artery shown by transesophageal echocardiography. Empty arrows indicate thromboembolic clots.

In ICU, her platelet counts largely increased from 15.0 to 263.0 (× 10^9^/L) and her coagulation parameters acutely aggravated to hypercoagulable state as assessed by thromboelastography (maximal amplitude of 122 mm). Our patient suffered acute circulatory collapse (systolic blood pressure [SBP], 53 mm Hg; diastolic blood pressure [DBP], 21 mm Hg), and exhibited a gasping breathing pattern indicative of respiratory distress and a decline in mental status from alert to stupor. The ICU attending physicians immediately performed resuscitation management consisting of aggressive fluid therapy, blood product transfusion (3 units of PRBCs [blood type O^+^]), high-dose vasopressor infusion and endotracheal intubation with mechanical ventilation. Although her vital signs and mental status recovered thereafter, the patient required emergency exploratory abdominal surgery to repair the vessel injury incurred during surgery. During surgery, bleeding from the site of collateral vascular injury near the pancreas was detected and repaired. However, hypotension (SBP, 65 mm Hg; DBP, 50 mm Hg), bradycardia (<40 beats/minutes) and arrhythmia developed, accompanied by a large increase in central venous pressure (up to 30 mm Hg). Rescue TEE, urgently performed to evaluate cardiac structure and function, incidentally identified multiple, huge thromboembolic clots in both atrial chambers and a left-shifted deviation of the atrial wall (Fig. 1). The large flapping thromboembolic clots on both cardiac valves severely impaired blood flow from the atrium to the ventricle (Fig. 2) and caused severe mitral (Fig. 3) and tricuspid regurgitation. Other large thromboembolic clots obstructed left ventricular outflow (Fig. 4) and compressed the right and left pulmonary arteries (Fig. 5), resulting in severe cardiac collapse. The patient therefore underwent emergency thromboembolectomy, including cardiopulmonary bypass and hypothermia therapy (body temperature, 34°C). Nonetheless, severe cardiac and respiratory distress continued, necessitating intraoperative venoarterial extracorporeal membrane oxygenation (VAECMO; maintained in the ICU). During emergency surgery, 25 units of PRBC and 5 units of FFP were transfused. In the ICU, despite fluid infusion, blood product transfusion (10 units of PRBCs) and high-dose vasopressor administration, our patient, while still on VAECMO, suffered severe hypotension and continued massive bleeding due to a deficiency and dilution of coagulation factors. The patient died 1 day after emergency surgery.

## 3 Discussion

ABO incompatibility, splenectomy and autoimmune hepatitis in our patient resulted in her being highly vulnerable to postoperative thrombosis. The acute imbalance between pro- and anti-coagulants eventually resulted in fatal intracardiac and pulmonary arterial thromboembolic damage. Although the patients hemodynamics and coagulation-related findings were clinically tolerable before and during ABOi LDLT, abrupt perioperative changes in coagulation features, including her platelet count, exacerbated the risk of early thromboembolism after ABOi LDLT with splenectomy for autoimmune hepatitis.

Thrombosis in the graft vessels occurs in 24% of ABOi LDLT recipients.^[12]^ Moreover, ABO incompatibility may adversely affect coagulation, including platelet count and function, because of plasmapheresis related to citrate load and metabolic disturbances.^[13]^ Although there are no limitations on the number of plasmapheresis treatments that can be applied, a titer reduction rate ([titer before treatment – titer directly before transplantation]/total number of treatments) below the titer increment (titer recovery between the end of plasmapheresis and the beginning of the next plasmapheresis) is associated with a risk of hepatic arterial thrombosis and graft failure.^[8]^ In the antibody-mediated immune response, recipient isohemagglutinins seem to facilitate inflammatory immune activation, including complement cascades, which may increase the risk of hepatic artery thrombosis due to enhanced interactions between platelets and von Willebrand factor.^[14]^ Because ABO antigen is expressed on the surfaces of red blood cells and on the biliary/vascular endothelium, blood antigens may still be expressed on the donor vascular endothelium postoperatively, resulting in an immunological thrombotic response in the hepatic vessels.^[15,16]^ In our patient (donor blood group A^+^ and recipient blood group B^+^), the target isoagglutinin titer of <1:8 was reached 4 times by plasmapheresis and the CD20 level was decreased (from 6.1%–0.1%) by rituximab administered before surgery. However, our patient required transfusion with large quantities of blood products due to plasmapheresis and hemorrhage, before and during surgery. Due to the immunologic complexity associated with transplantation across ABO blood barriers,^[17,18]^ our patient suffered major thrombotic complications during the acute recovery phase after surgery.

Hypersplenism related to the sequestrations of blood cellular components has been reported in 24% of patients with cirrhosis.^[19]^ Thrombocytopenia is the most clinically relevant sign of hypersplenism and can increase the bleeding tendency in this population.^[20]^ A platelet count <50 × 10^9^/L may contribute to large-volume blood loss during invasive procedures, such that preemptive treatment, including surgical removal of the enlarged spleen, should be considered.^[21]^ In our patient, the platelet count and aggregability were markedly suppressed before splenectomy, but both increased significantly during the immediate postoperative period.^[22]^ In ABOi LDLT, simultaneous splenectomy is frequently performed to ameliorate portal hypertension and pancytopenia (particularly, thrombocytopenia), and improve the immune status.^[23]^ However, a major complication of splenectomy is thrombosis in the splenic and/or portal vein, such that the thrombotic risk is highest within the first week after surgery.^[24]^ Following splenectomy, recipient findings including an increased platelet count and decreased portal flow indicate an elevated risk of portal vein thrombosis.^[25,26]^ In our patient, the platelet count increased dramatically immediately after splenectomy (from 17.0 × 10^9^/L 1 day preoperatively to 263.0 × 10^9^/L immediately after surgery, as measured in the ICU). In the ICU, the patient suffered abrupt and severe circulatory and respiratory distress, which resulted in an altered mental state 1 day after ABOi LDLT. She therefore required intensive resuscitation therapy and emergency reoperation. Acute disruption of rebalanced hemostasis may play a role to trigger severe hemostatic activation.^[27]^ The multiple, huge thromboembolic clots in all 4 cardiac chambers and pulmonary artery of our patient led to her death.

Autoimmune hepatitis is one of the major indications for LT; its etiology is unknown but it is mostly seen in middle-aged female patients.^[18]^ Patients with autoimmune hepatitis are at high risk of developing hepatic vascular thrombosis during the perioperative period. Thrombosis occurs in 2% to 26% of patients waiting for LT, and the incidence increases to 50% during LT.^[28]^ Previous studies reported that autoimmune hepatitis is significantly associated with thrombotic complications and disorders such as antiphospholipid syndrome. They also showed that patients with autoimmune hepatitis have a higher prevalence of anticardiolipin antibody than those with other liver diseases, although a higher proportion of anticardiolipin antibodies is not related to the clinical development of anticardiolipin syndrome.^[29–31]^ A study by Elefsiniotis et al^[32]^ found that chronic infection with hepatitis B virus may stimulate the production of anticardiolipin antibody, and that patients with hepatitis B who are positive for anticardiolipin antibody are more prone to developing portal vein thrombosis than those who are negative for the antibody. Our patient suffered refractory autoimmune hepatitis and was a hepatitis B carrier. Although the levels of autoimmune biomarkers, such as anticardiolipin antibody, were not measured before or after surgery, her prolonged exposure to autoimmune antibodies and hepatitis B virus may have resulted in a prothrombotic environment.

Our case demonstrates the importance of monitoring and controlling the risk factors for thromboembolism, such as hemorrhage, hypotension, and stagnant blood flow, during the peak period of reactive thrombocytosis after ABOi LDLT with splenectomy for autoimmune hepatitis. The appropriate use of anticoagulants or inhibitors of platelet aggregation is recommended as necessary. The patency of systemic and hepatic vascular flows, and the levels of autoimmune biomarkers such as anticardiolipin antibody, should be determined during the perioperative period, when the patients immunologic vulnerability and risk of complications are high.

## Author contributions

**Conceptualization:** Min Suk Chae.

**Data curation:** Won Kyu Choi, Junghan Kim, Ho Joong Choi, Sang Hyun Hong, Min Suk Chae.

**Formal analysis:** Won Kyu Choi, Ho Joong Choi, Sang Hyun Hong, Min Suk Chae.

**Investigation:** Min Suk Chae.

**Supervision:** Min Suk Chae.

**Visualization:** Min Suk Chae.

**Writing – original draft:** Won Kyu Choi, Min Suk Chae.

**Writing – review & editing:** Min Suk Chae.

## Abbreviations

Abbreviations: ABOi LDLT = ABO incompatible living donor liver transplantation, CD = cluster of differentiation, CVP = central venous pressure, DBP = diastolic blood pressure, FFP = fresh frozen plasma, ICU = intensive care unit, INR = international normalized ratio, PRBC = packed red blood cells, SBP = systolic blood pressure, SDP = single donor platelets, TEE = transesophageal echocardiography, VAECMO = venoarterial extracorporeal membrane oxygenation.
